# Chordin-Like 1 Regulates Epithelial-to-Mesenchymal Transition and Metastasis *via* the MAPK Signaling Pathway in Oral Squamous Cell Carcinoma

**DOI:** 10.3389/fonc.2022.862751

**Published:** 2022-04-14

**Authors:** Qiuyu Wu, Zhichao Zheng, Junwei Zhang, Zhengguo Piao, Mengyu Xin, Xi Xiang, Antong Wu, Tianyu Zhao, Songkai Huang, Yu Qiao, Jiayu Zhou, Shaofen Xu, Haoyu Cheng, Lihong Wu, Kexiong Ouyang

**Affiliations:** ^1^ Department of Oral and Maxillofacial Surgery, Affiliated Stomatology Hospital of Guangzhou Medical University, Guangzhou Key Laboratory of Basic and Applied Research of Oral Regenerative Medicine, Guangzhou, China; ^2^ Jiangmen Central Hospital, Affiliated Jiangmen Hospital of Sun Yat-sen University, Jiangmen, China; ^3^ Affiliated Stomatology Hospital of Guangzhou Medical University, Guangzhou Key Laboratory of Basic and Applied Research of Oral Regenerative Medicine, Guangzhou, China

**Keywords:** chordin-like 1, oral squamous cell carcinoma (OSCC), epithelial-to-mesenchymal transition (EMT), MAPK, metastasis

## Abstract

**Background:**

Accumulating evidence suggests that dysregulation of Chordin-like 1 (CHRDL1) is associated with malignant biological behaviors in multiple cancers. However, the exact function and molecular mechanism of CHRDL1 in oral squamous cell carcinoma (OSCC) remain unclear.

**Methods:**

The expression levels of CHRDL1 in OSCC tissues and CAL27 cells were determined by RT-qPCR. Immunohistochemical staining was applied to detect CHRDL1 protein expression in sample tissues from OSCC patients. Gain of function and knockdown by lentivirus were further used to examine the effects of CHRDL1 on cell proliferation, migration, invasion, and adhesion in OSCC. Tail vein injection of CAL27 cells with dysregulated CHRDL1 expression was further used to examine the effect of CHRDL1 on lung colonization. RNA sequencing was performed to explore the molecular mechanisms of CHRDL1 that underlie the progression of OSCC.

**Results:**

CHRDL1 was significantly downregulated in OSCC tissues and CAL27 cells compared to controls. CHRDL1 knockdown enhanced migration, invasion, adhesion, and EMT, but not proliferation, in CAL27 cells. Overexpression of CHRDL1 had the opposite effects. Moreover, CHRDL1 was proven to inhibit tumor metastasis *in vivo*. Mechanistically, MAPK signaling pathway components, including ERK1/2, p38, and JNK, were found to regulate the malignant biological behaviors of CAL27 cells.

**Conclusions:**

Our results suggest that CHRDL1 has an inhibitory effect on OSCC metastasis *via* the MAPK signaling pathway, which provides a new possible potential therapeutic target against OSCC.

## Introduction

Oral squamous cell carcinoma (OSCC) is one of the most common lethal malignancies worldwide, with poor prognosis (1). It is reported that approximately 377,713 cases of oral cancer are newly diagnosed each year, accounting for approximately 2% of all malignancies reported worldwide. The latest data indicate that the overall number of cases will even rise to 510,948 in 2035 (2). The traditional treatments for OSCC are surgical resection, radiotherapy, or chemotherapy or their combination. Although reconstructive surgery of the lesion is always applied to restore appearance and function, the side effects of surgical resection, radiotherapy, or chemotherapy persistently and irreversibly reduce patient quality of life. Recent research has shown an improvement in the survival rates of OCSS patients from 55% in 1986 to 60% in 2003 (3). However, the improvement in the survival rate of OSCC has been much smaller than that for other cancer types, such as breast cancers and colon cancers. It is widely accepted that the accumulation of genetic alterations in oncogenes and tumor suppressors leads to the emergence of OSCC (4, 5). The potential pathogenesis, including the progression mechanism, of OSCC is not fully understood.

Bone morphogenetic proteins (BMPs), belonging to the TGF-β family (6), play an important role in early embryonic development and homeostasis, including bone formation and regeneration (7). Recently, several studies have indicated that several BMPs, such as BMP-2, BMP-4, BMP-6, and BMP-7, are also involved in tumor development and progression (8, 9). Studies have shown that BMP-2 has a positive effect on the invasion ability of oral, gastric, breast, colon, bladder, and pancreatic cancers (10–12). BMP-2 induced the invasion of OSCC cells, possibly through CCL5 release, in coculture models (11). BMP-9 was reported to stimulate the proliferation of ovarian cancer cells (13). Rothhammer et al. demonstrated that BMP-4 promotes melanoma cell invasion and migration (14). In OSCC, BMP-7 was found to be the key to the acquisition of cetuximab resistance (15).

Compared with the fully elucidated role of BMPs in tumors, research on their antagonists in tumors is relatively limited. Chordin like-1 (CHRDL1), a BMP antagonist, was originally reported to be expressed in the developing retina (16). Mutation of CHRDL1 is related to X-linked megalocornea, an ocular anterior segment disorder (17–20). Recent studies have associated CHRDL1 with various cancers, including breast cancer (21–24), lung cancer (25–28), gastric cancer (29, 30), thyroid cancer (31–34), T-cell acute lymphoblastic leukemia (T-ALL) (35), melanoma (36), and OSCC (37). CHRDL1 was proven to block BMP-induced increases in breast cancer cell migration and invasion. High CHRDL1 expression is associated with better clinical outcomes in patients with breast cancer (21). Furthermore, CHRDL1 expression is significantly downregulated in gastric cancer tissues and associated with poor survival, and CHRDL1 knockdown promotes tumor cell proliferation and migration through BMPR II by activating Akt, Erk, and β-catenin (29). In melanoma, CHRDL1 showed growth-suppressing properties in melanoma-derived cell lines with DNA methylation and genomic deletion (36). However, most of these studies provided only correlation analyses of CHRDL1 with different tumors. The specific role of CHRDL1 in cancers should be further explored. Moreover, the function and regulatory mechanism of CHRDL1 in OSCC have not been studied.

Here, we aimed to identify the role of CHRDL1 in OSCC and to explore the relevant mechanisms, which may provide a new potential target for OSCC therapy.

## Methods and Materials

### Clinical Specimen Collection and Immunohistochemistry Staining for CHRDL1

The studies involving human participants were approved by the Ethics Committee of the Stomatology Hospital of Guangzhou Medical University (Approval No. KY2019026). Thirty primary OSCC specimens and adjacent tissue specimens were obtained from the Department of Oral and Maxillofacial Surgery, Stomatology Hospital of Guangzhou Medical University. All OSCC specimens were pathologically diagnosed as OSCC, and the adjacent tissue specimens were obtained ≥2 cm from the resection margin, which were pathologically diagnosed to have no precancerous or reactive changes histologically. All specimens were frozen immediately in liquid nitrogen after resection.

Immunohistochemical staining (IHC) was performed on 5 human OSCC tumor tissues and paired adjacent specimens. Paraffin-embedded tissues were cut into 4-µm sections. The samples were dewaxed, dehydrated, and then treated with 3% H_2_O_2_ solution to inhibit the endogenous peroxidase activity. Antigen retrieval was carried out by microwave heating in sodium citrate buffer (pH 7.8). Slides were then blocked with 5% BSA solution. Tissue sections were further incubated with primary antibody against CHRDL1 (1:150 dilution, Zen-Bio) at 4°C for 12 h and then washed and incubated with secondary antibody (1:100 dilution, ZSJB-Bio) at 37°C for 1 h. Finally, slides were incubated with diaminobenzidine (DAB, ZSJB-Bio) and counterstained with hematoxylin.

IHC slices were scanned by a digital tissue slice scanner (3D HISTECH, Magyarország). The Servicebio image analysis system was used to score the slides for positivity. Positivity grading was first performed (I) as follows: negative without staining, 0 points; weak positive/light yellow, 1 point; medium positive/brownish yellow, 2 points; strong positive/brown, 3 points; then, the cumulative weak, medium, and strong positive area, tissue area, and integrated optical density (IOD) value of positive in the measurement area were calculated. The histochemistry score (H-score) is the output of a histological scoring method for immunohistochemistry. The number of positive cells in each section and their staining intensity were converted into corresponding values for semiquantification of the tissue staining, as follows: H-Score = ∑(pi×i) = (percentage of weak intensity area ×1) + (percentage of moderate intensity area ×2) + (percentage of strong intensity area ×3), where pi represents the percentage of the pixel area of positive signal, and i represents positive grade (38, 39).

### Cell Line Acquisition and Culture

The human tongue squamous cell carcinoma cell line CAL27 (ATCC, ATCC^®^ CRL-2095™) was purchased from ATCC, while the normal oral epithelial keratinocyte line HOK was purchased from AULU (Guangdong, China). CAL27 and HOK cells were cultured in Dulbecco’s modified Eagle’s medium (DMEM) (Gibco, Waltham, MA, USA). The complete cell culture medium contained 10% fetal bovine serum (FBS, Gibco) and 1% penicillin/streptomycin (Gibco). The serum-free cell culture medium for carcinoma cells was prepared as DMEM containing 1% penicillin/streptomycin for a number of subsequent experiments (e.g., wound-healing assay, cell migration, and invasion assays). The cells were cultured in a humidified incubator at 37°C and 5% CO_2_ incubator.

### Overexpression/Knockdown of CHRDL1 in CAL27 by Lentiviral Transfection

Commercial lentivirus (lenti-CHRDL1 and shRNA-CHRDL1; OBIO, Shanghai, China) was utilized to overexpress or silence CHRDL1. An empty carrier lentivirus (lenti-NC/shRNA-NC) was used as a negative control. CAL27 cells were infected with the lentivirus (MOI = 60) and screened with puromycin (2 µg/ml) for 15 days. Quantitative reverse transcription-polymerase chain reaction (RT-qPCR) and Western blotting (WB) were used to validate the overexpression/knockdown level of CHRDL1.

### RNA Isolation and Quantitative Real-Time PCR

Total RNA from the clinical specimens was extracted using TRIzol Reagent (Invitrogen, USA) according to the manufacturer’s instructions. Total RNA from the cells was extracted using an RNA extraction kit (19221ES50, Yeasen, China). The quantity and quality of RNAs were detected by A260/A280 with a spectrophotometer (NanoDrop 2000, Thermo Fisher Scientific, Waltham, MA, USA). Two micrograms of total RNA was used to synthesize cDNA (11141ES60, Yeasen, China). RT-qPCR was performed using a SYBR Green qPCR kit (11199ES08, Yeasen, China). Relative mRNA expression was normalized to that of the internal GAPDH control. The primer sequences are listed in **Supplementary Table S1**. The relative expression of targeted genes was calculated by the 2^−ΔΔCt^ method. Each test was repeated at least three times.

### Western Blot Analysis

Total proteins were isolated from cell samples through precooled cell lysis buffer (Cell Signaling Technology, Danvers, MA, USA) with protease inhibitor and phosphatase inhibitors (RayBiotech, Guangzhou, China) and quantified by a BCA protein assay kit (SE248351, Thermo Fisher, USA). Equal protein extracts (30 µg) were separated by SDS–PAGE (PG112, Epizyme, China) and transferred to polyvinylidene fluoride membranes (Millipore, Burlington, MA, USA). After incubation with the primary antibody and the secondary antibody, the target protein was visualized by chemiluminescence using an ECL kit (P001AS, Beyotime, China). The antibodies used in the Western blot assay are listed as follows: CHRDL1 (1:500, Thermo Fisher, USA), Fibronectin (1:1,000, Proteintech, China), E-cadherin (1:1,000, Proteintech, China), E-cadherin (1:1,000, Proteintech, China), Cytokeratin 18 (1:1,000, Abcam, UK), β-actin (1:1,0000, Cell Signaling Technology, USA), JNK (1:1,000, Cell Signaling Technology, USA), p-JNK (1:1,000, Cell Signaling Technology, USA), ERK1/2 (1:1,000, Cell Signaling Technology, USA), p-ERK1/2 (1:1,000, Cell Signaling Technology, USA), p38 (1:1,000, Cell Signaling Technology, USA), p-p38 (1:1,000, Cell Signaling*nbsp;Technology, USA), and goat anti-rabbit IgG H&L (HRP) antibody (1:10,000, Abcam, UK). β-actin served as the internal control to calculate the relative expression of the targeted proteins.

### Wound-Healing Assay

For the wound-healing assay, transfected CAL27 cells were first incubated and cultured with complete medium. A scratch was made after a confluent monolayer of cells was formed. Afterward, the cells were washed with phosphate-buffered saline (PBS) and cultured with serum-free medium. Images were taken at 0 and 24 h after wound generation. The wound-healing areas were assessed by ImageJ to calculate the wound-healing rate. Wound-healing rate% = [Area at 0 – Area at 1]/Area at 0 × 100% (Area at 0 is the area of the wound measured immediately after scratching, and Area at 1 is the area of the wound measured at 1 h after scratching). The wound area was measured at 0 and 24 h. Each test was repeated at least three times.

### Cell Migration Assay

CAL27 cells were seeded at a density of 1 × 10^5^ in serum-free DMEM in the upper wells of Transwell chambers (8 μm pore size, Corning, New York, NY, USA), while the lower wells were filled with complete medium containing 20% FBS. After 48 h, cells in the upper layer were removed with a swab. The cells on the bottom membrane were fixed with 4% paraformaldehyde and stained with crystal violet. Five random visual fields were photographed for each group, and the experiments were repeated three times.

### Cell Invasion Assay

CAL27 cells were seeded at a density of 2 × 10^5^ in serum-free DMEM in the upper wells of chambers that were coated with 60 µl of Matrigel (200 μg/µl, Corning) and incubated for 2 h at 37°C, while the lower wells were filled with complete medium containing 20% FBS. Cells in the upper layer were removed with a swab after 36 h of culture, and cells on the bottom membrane were fixed with 4% paraformaldehyde and stained with crystal violet. Each group was photographed in five randomized visual fields, and the experiments were repeated three times.

### Cell Adhesion Assay

A Cell Adhesion Detection kit (BB-48120, BestBio Science, China) was used according to the manufacturer’s instructions to analyze the adhesion ability of CAL27 cells. Briefly, the coating buffer (100 µl) was transferred to each well in a 96-well plate and incubated overnight at 4°C. Cells (5×10^4^) were inoculated into each well of the coated 96-well plate and allowed to adhere for 1.5 h at 37°C. After the cells were washed three times with PBS, cell imaging was performed. The optical density value at 450 nm was measured to calculate the cell adhesion rate. Each test was repeated at least three times.

### Rhodamine-Conjugated Phalloidin Staining and Confocal Microscopy

CAL27 cells were seeded at a density of 1,000 in a confocal dish. After 24 h, cell monolayers were fixed with 4% paraformaldehyde and stained with rhodamine-conjugated phalloidin (40737ES75, Yeasen, China) according to the manufacturer’s instructions. Confocal images were captured using a confocal laser scanning microscope (LEIKATCSSP8, Leica, Germany).

### mRNA Sequencing

Total RNA was extracted from transfected CAL27 cells (OV-CHRDL1/OV-NC and sh1-CHRDL1/sh-NC) using TRIzol reagent (Invitrogen Life Technologies, USA) following the manufacturer’s protocol. RNA concentration and purity were checked with a Nanodrop 2000 instrument (Thermo Fisher Scientific, USA). After RNA quality control was performed, the libraries for next-generation sequencing were prepared using the TruSeqTM RNA Sample Prep Kit (Illumina, USA) according to the manufacturer’s instructions. Sequencing was performed by Shanghai Origingene Biopharm Technology Co., Ltd. (Shanghai, China). After quality control of the original data, the high-quality sequencing data were compared with the designated reference genome. The expression values were calculated by the StringTie tool, and the tDESeq algorithm was applied to filter the differentially expressed genes. Gene Ontology (GO) and Kyoto Encyclopedia of Genes and Genomes (KEGG) analyses were performed to reveal the involved pathways.

### Lung Metastasis in Nude Mice

The animal experiments were conducted in accordance with the guidelines approved by the Institutional Animal Care and Use Committee of the First Affiliated Hospital of Guangzhou Medical University of China (Approval number was 2017-086).

Four-week-old male BALB/c-nude mice were purchased from GemPharmatech (Jiangsu, China). All animal experiments were carried out following the guidelines set by the Institutional Animal Care and Use Committee of the First Affiliated Hospital of Guangzhou Medical University of China. Nude mice were randomly divided into four groups. CAL27 cells (1×10^6^) were suspended in 0.1 ml PBS and injected *via* the tail vein into animals that had been anesthetized with 3% isoflurane. After 9 weeks, the nude mice were anesthetized and euthanized by cervical dislocation. Lungs were collected, weighed, rinsed, fixed, and embedded in paraffin blocks and sectioned for H&E staining to confirm metastasis. Three slices were acquired from each lung sample. The number of pulmonary tumor nodules were counted under the dissecting microscope. The averaged lung colonies from 3 slices were calculated for each lung sample.

### Cell Proliferation Assay

Cells (4,000) were cultured in 96-well plates, and cell proliferation was detected using a Cell Counting Kit-8 (Dojindo, Kumamoto, Japan) at 1, 3, and 5 days of culture according to the manufacturer’s instructions. EdU staining was performed to assess cell proliferation ability by the Cell-Light EdU imaging kit (C0071S, Beyotime, China) following the manufacturer’s instructions. A colony formation assay was performed. One thousand transfected CAL27 cells were seeded in six-well plates and cultured for 8 days in complete medium. The colonies were further fixed with 4% paraformaldehyde and stained with crystal violet. Colonies that contained more than 50 cells were counted. Each test was repeated at least three times.

### Statistical Analysis

The data were analyzed using statistical software (SPSS Statistics 16.0). The data are presented as the means and standard deviations. Student’s *t*-test was used for comparisons of normally distributed data. The Mann–Whitney *U* test was used for nonnormally distributed data. A *p*-value <0.05 was considered statistically significant.

## Results

### CHRDL1 Is Downregulated in OSCC Both *In Vivo* and *In Vitro*

**Figure 1:**
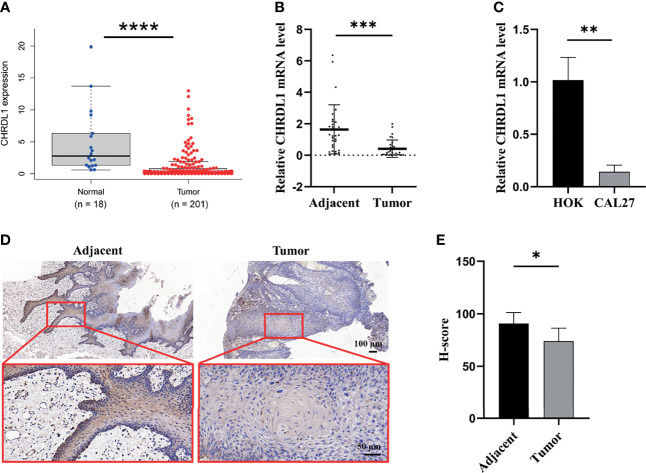
The expression of CHRDL1 was downregulated in OSCC tissue and cell lines. **(A)** Relative mRNA expression of CHRDL1 in OSCC tissues (*n* = 201) and normal tissues (*n* = 18) from the TCGA database. **(B)** The relative mRNA expression of CHRDL1 in 30 specimens of OSCC and adjacent tissues was determined by qRT-PCR. **(C)** The relative mRNA expression of CHRDL1 in normal human oral keratinocytes (HOK) and the OSCC cell line CAL27 was determined by RT-qPCR. CHRDL1 mRNA expression was normalized to GAPDH. **(D, E)** IHC analysis of CHRDL1 in tumor and adjacent tissues (*n* = 5). Error bars represent the standard deviation. *p < 0.05, **p < 0.01, ***p < 0.001, ****p < 0.0001.

The data from the Gene Expression Profiling Interactive Analysis database indicated that the expression of CHRDL1 was downregulated in HNSC tissues compared to normal tissues (**Figure 1A**). We further collected samples from 30 OSCC patients, and qRT-PCR indicated that the expression of CHRDL1 was significantly decreased in OSCC tissues compared to adjacent tissues (**Figure 1B**). A lower expression level of CHRDL1 was also detected in the common OSCC cell line CAL27 than in the normal cell line HOK (**Figure 1C**). IHC analysis of tissue samples showed that CHRDL1 was downregulated in OSCC tissues. Representative IHC images revealed that CHRDL1 protein was primarily distributed in the epithelial structure of OSCC tissues and adjacent tissues. However, there was little staining of CHRDL1 in either the tumor stroma in OSCC or subepithelial tissues in the adjacent normal tissues (**Figures 1D, E**). These data demonstrated that CHRDL1 was typically negatively correlated with OSCC development.

### Overexpression of CHRDL1 Inhibits Malignant Biological Behaviors of CAL27 Cells

**Figure 2:**
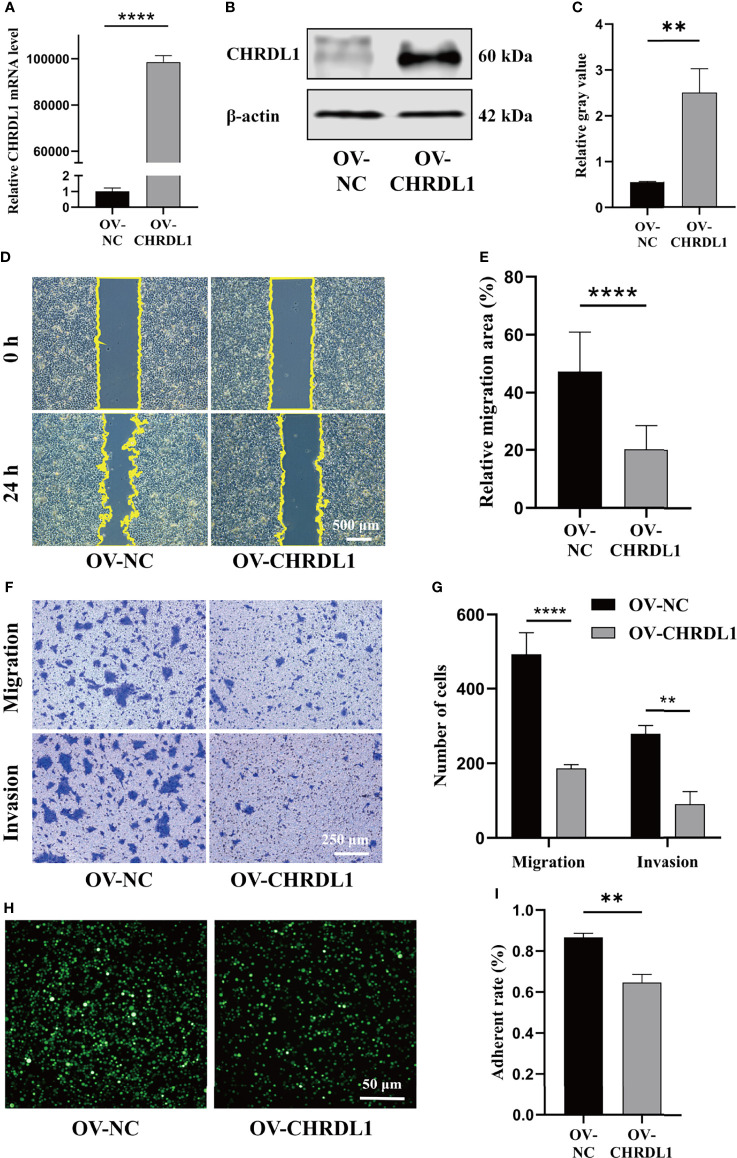
Overexpression of CHRDL1 inhibits CAL27 cell migration, invasion, and adhesion. **(A)** Relative mRNA expression of CHRDL1 in CAL27 cells after lentivirus transfection. **(B, C)** CHRDL1 protein expression in CAL27 cells after lentivirus transfection was identified by Western blot and quantitatively analyzed. **(D, E)** Representative images of wound-healing assays of transfected CAL27 cells at 0 and 24 h and statistical results from each group. **(F, G)** Migration and invasion assays of transfected CAL27 cells. Representative images of cell migration and invasion in each group are shown in **(F)**. Statistical analyses of cell migration and invasion are shown in **(G)**. **(H, I)** Cell adhesion assay of transfected CAL27 cells. Representative fluorescence images from each group **(H)** and statistical analyses of the adherence rate **(I)**. Error bars represent the standard deviation. **p < 0.01, ***p < 0.001.

The role of CHRDL1 in the progression of OSCC was explored in the CAL27 cell line. CHRDL1 was overexpressed in the CAL27 cell line by lentivirus infection (**Figures 2A–C**). Overexpression of CHRDL1 markedly suppressed the wound-healing and migration abilities of CAL27 cells (**Figures 2D–G**). Furthermore, CHRDL1 overexpression significantly decreased the number of invading CAL27 cells (**Figures 2F, G**). The cell adhesion assay showed a lower adhesion rate in the CHRDL1 overexpression (OV-CHRDL1) group than in the control group (NC-CHRDL1) (**Figures 2H, I**). However, the EdU assay, colony formation assay, and CCK-8 assay showed no significant difference between the OV-CHRDL1 group and the NC-CHRDL1 group, indicating that overexpression of CHRDL1 did not affect the proliferation of CAL27 cells (**Supplementary Figure 1**). These results suggested that upregulation of CHRDL1 inhibited metastasis rather than proliferation in the CAL27 cell line.

### CHRDL1 Knockdown Promotes Malignant Biological Behaviors in CAL27 Cells

**Figure 3:**
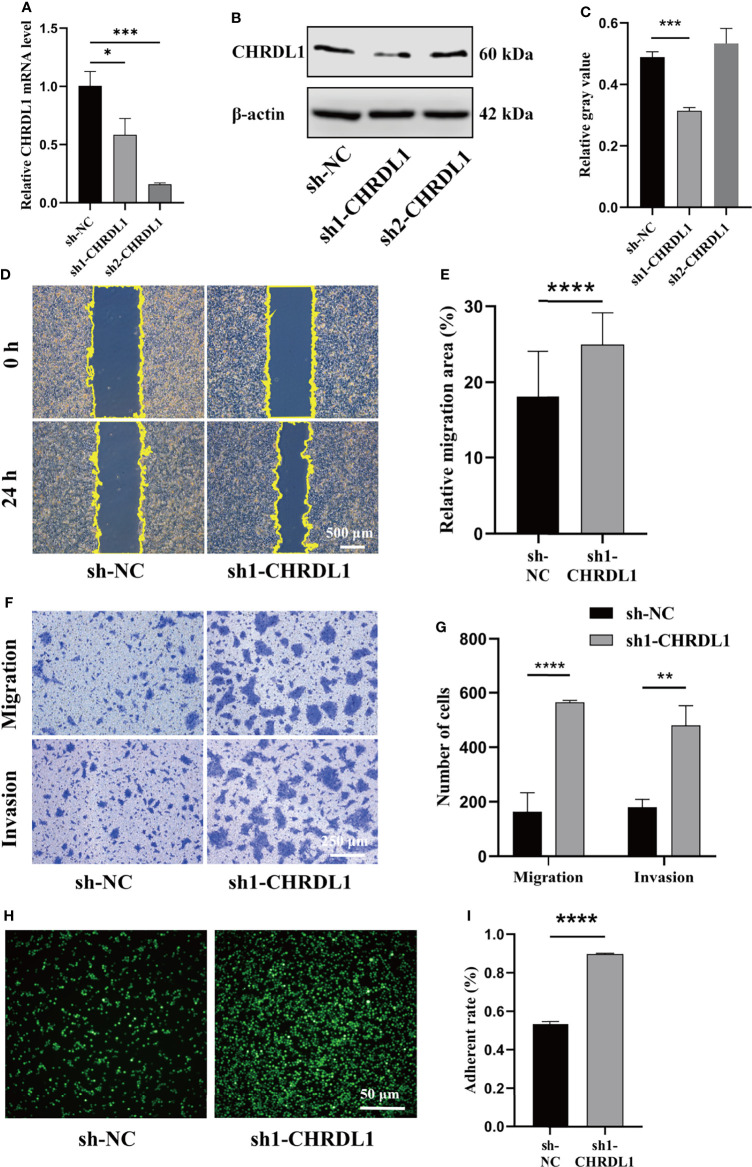
CHRDL1 knockdown promotes CAL27 cell migration, invasion, and adhesion. **(A)** Relative mRNA expression of CHRDL1 in CAL27 cells after lentivirus transfection. **(B, C)** CHRDL1 protein expression in CAL27 cells after lentivirus transfection was evaluated by Western blot and quantitatively analyzed. **(D, E)** Representative images of wound-healing assays of transfected CAL27 cells at 0 and 24 h and statistical analyses of each group. **(F, G)** Migration and invasion assays of transfected CAL27 cells. Representative images of cell migration and invasion in each group are shown in **(F)**. Statistical analyses of cell migration and invasion are shown in **(G)**. **(H, I)** Cell adhesion assay of transfected CAL27 cells. Representative fluorescence images of each group **(H)** and statistical analyses of the adherence rate **(I)**. Error bars represent the standard deviation. *p < 0.05, **p < 0.01, ***p < 0.001, ****p < 0.0001.

To determine whether silencing CHRDL1 promoted malignant biological behaviors in the CAL27 cell line, two stable CHRDL1-knockdown CAL27 cell lines (sh1-CHRDL1 and sh2-CHRDL1) and their control group (sh-NC) were established. Although CHRDL1 mRNA levels were significantly decreased in both stable CHRDL1-knockdown cell lines (**Figure 3A**), the protein level was reduced only in the sh1-CHRDL1 cell line and was obviously increased in the sh2-CHRDL1 cell line (**Figure 3B, C**). Thus, the sh1-CHRDL1 CAL27 cell line was used for the following experiments. We demonstrated that CHRDL1 silencing markedly promoted the wound-healing and migration abilities of CAL27 cells (**Figures 3D–G**). The sh1-CHRDL1 CAL27 cells also exhibited enhanced invasion (**Figures 3D–G**) and adhesion ability (**Figures 3H, I**) compared to sh-NC CAL27 cells. Intriguingly, proliferation assays also showed no significant difference between the sh1-CHRDL1 group and the sh-NC group (**Supplementary Figure 1**). These findings confirmed that CHRDL1 silencing promoted the progression of CAL27 cells.

### CHRDL1 Knockdown Induces the Epithelial–Mesenchymal Transition Phenotype in CAL27 Cells

**Figure 4:**
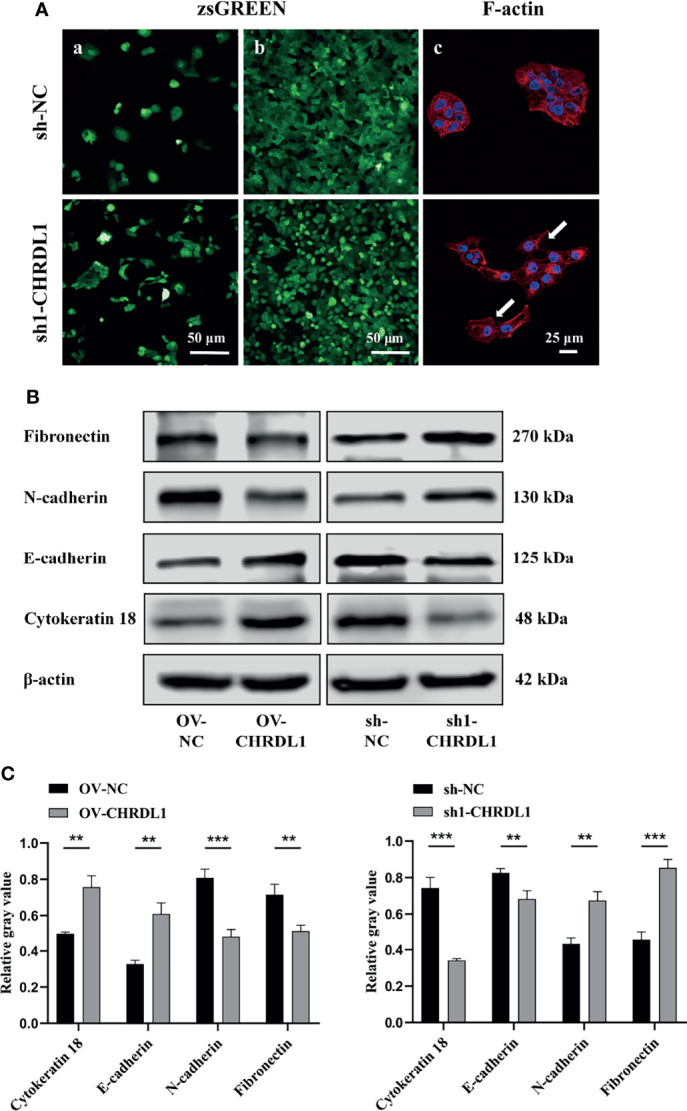
Depletion of CHRDL1 induces the EMT phenotype in CAL27 cells. **(A)** Representative fluorescence images of sh-NC/sh1-CHRDL1 CAL27 cells under low **(A)** and high (b) cell density conditions. (c) Rhodamine-conjugated phalloidin staining of F-actin in sh-NC/sh1-CHRDL1 CAL27 cells, examined using confocal microscopy. The arrows indicate filopodia. **(B, C)** The expression of EMT markers (Fibronectin, N-cadherin, E-cadherin, and Cytokeratin 18) in CAL27 cells after lentivirus transfection was detected by Western blot and quantitatively analyzed. All experiments were performed three times, and one representative experiment is shown. Error bars represent the standard deviation. **p < 0.01, ***p < 0.001.

EMT has been widely reported to increase OSCC metastasis (40, 41). Compared to sh-NC cells, sh1-CHRDL1 cells became fusiform and fibroblast-like in shape and lost their polygonal epithelial morphology (**Figure 4A** a). The sh1-CHRDL1 CAL27 cells also became more dispersed and showed loose cell-to-cell contact compared to sh-NC CAL27 cells under high cell density conditions (**Figure 4A** b). Rhodamine phalloidin staining was further performed to detect F-actin. Actin-rich membrane protrusions called filopodia (white arrow) were clearly seen at the periphery of sh1-CHRDL1 CAL27 cells but not sh-NC CAL27 cells (**Figure 4A** c). To further examine the EMT-like phenotype of sh1-CHRDL1 CAL27 cells, the expression of epithelial and mesenchymal markers, including cytokeratin 18, E-cadherin, N-cadherin, and fibronectin, was examined by WB (**Figures 4B, C**). The protein expression levels of E-cadherin and cytokeratin 18, epithelial differentiation markers, were significantly upregulated in OV-CHRDL1 CAL27 cells compared to OV-NC CAL27 cells. The protein expression levels of the mesenchymal differentiation markers N-cadherin and fibronectin were significantly downregulated in OV-CHRDL1 CAL27 cells compared to OV-NC CAL27 cells. In contrast, the protein expression levels of E-cadherin and cytokeratin 18 were 1.21- and 2.17-fold downregulated, respectively, in sh1-CHRDL1 CAL27 cells compared to sh-NC CAL27 cells, while the protein expression levels of N-cadherin and fibronectin were 0.64- and 0.53-fold upregulated, respectively, in sh1-CHRDL1 CAL27 cells compared to sh-NC CAL27 cells. These results suggested that CHRDL1 silencing in CAL27 cells led to the EMT phenotype, while overexpression of CHRDL1 in CAL27 cells inhibited the process. Thus, CHRDL1 knockdown may induce metastasis in CAL27 cells by activating EMT.

### CHRDL1 Regulates the MAPK Signaling Pathway in CAL27 Cells

**Figure 5:**
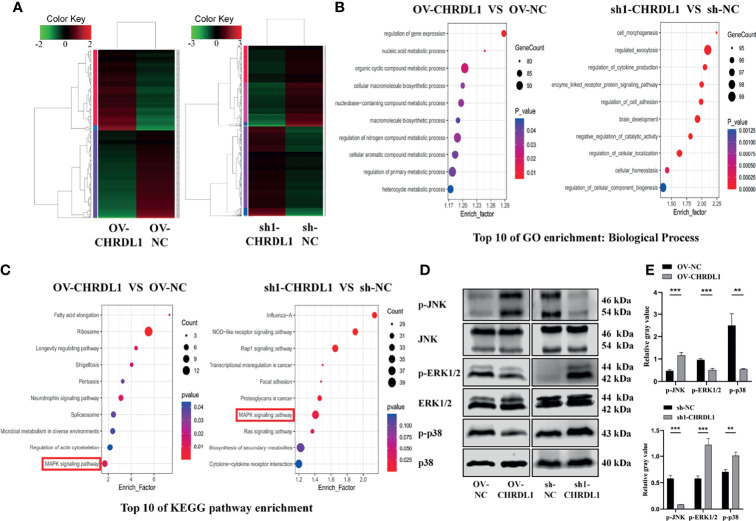
CHRDL1 regulates the MAPK signaling pathway in CAL27 cells. **(A)** Heatmap of differentially expressed genes between OV-CHRDL1 and OV-NC (left) and between sh1-CHRDL1 and sh-NC (right). Green indicates downregulated genes, and red indicates upregulated genes. The thresholds for up- and downregulation were a +2.0-fold and -2.0-fold change, respectively, and a *p*_adj_ < 0.05. **(B)** Top 10 GO-BP terms enriched among DEGs from OV-CHRDL1 CAL27 cells and sh1-CHRDL1 CAL27 cells. **(C)** Top 10 KEGG pathways enriched among DEGs from OV-CHRDL1 CAL27 cells and sh1-CHRDL1 CAL27 cells. **(D, E)** Protein expression of MAPK signaling pathway-related markers in CAL27 cells after lentivirus transfection. Error bars represent the standard deviation. **p < 0.01, ***p < 0.001.

To identify genes differentially expressed specifically in the context of high versus low CHRDL1 levels, RNA sequencing was performed in OV-NC/OV-CHRDL1 and sh-NC/sh1-CHRDL1 CAL27 cells. A fold-change >2.0 or <−2.0 and *p*_adj_ < 0.05 were chosen as cutoff criteria and visualized using the heatmap. A total of 163 upregulated and 179 downregulated genes in OV-CHRDL1 CAL27 cells compared to OV-NC cells were identified by gene expression analysis (**Figure 5A**, left). In addition, 1067 genes were upregulated and 952 genes were downregulated in sh1-CHRDL1 CAL27 cells compared to sh-NC cells (**Figure 5A**, right). Then, Gene Ontology (GO) and Kyoto Encyclopedia of Genes and Genomes (KEGG) enrichment analyses were performed on the differentially expressed genes (DEGs) identified in OV-CHRDL1 CAL27 cells and sh1-CHRDL1 CAL27 cells. The top 10 most significant GO biological processes (BP) are shown in **Figure 5B**. Cell morphogenesis and regulation of cell adhesion were identified in sh1-CHRDL1 CAL27 cells, consistent with the *in vitro* results. The top 10 enriched KEGG pathways are shown in **Figure 5C**. Interestingly, MAPK signaling was enriched in both OV-CHRDL1 CAL27 cells and sh1-CHRDL1 CAL27 cells. Thus, MAPK signaling pathway components were further probed by WB (**Figures 5D, E**). The protein expression of phosphorylated JNK (p-JNK) was upregulated, and that of phosphorylated ERK (p-ERK1/2) and p38 (p-p38) was downregulated, in OV-CHRDL1 CAL27 cells compared to OV-NC CAL27 cells. In contrast, the protein expression of p-JNK was downregulated and that of p-ERK and p-p38 was upregulated in sh1-CHRDL1 CAL27 cells compared to sh-NC CAL27 cells. These results indicated that CHRDL1 may influence the MAPK pathway in CAL27 cells to regulate cell metastasis.

### CHRDL1 Regulates Tumor Metastasis *In Vivo*

**Figure 6:**
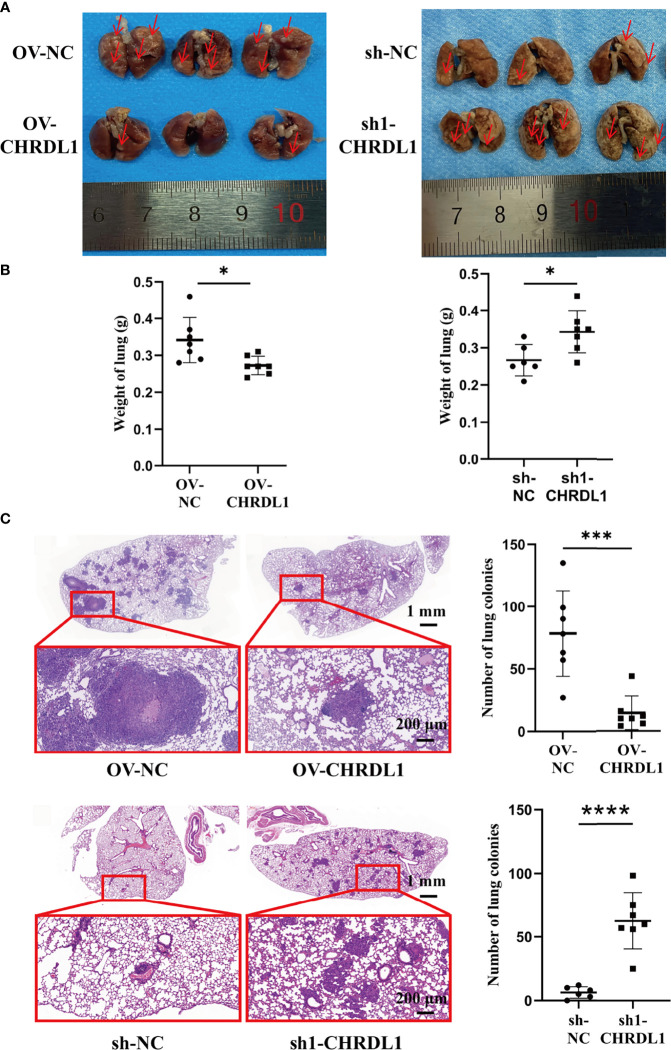
Effects of CHRDL1 on CAL27 cells *in vivo*. **(A)** Representative images of the lungs of BALB/c nude mice at 9 weeks after inoculation with OV-NC/OV-CHRDL1 (left) or sh-NC/sh1-CHRDL1 (right) CAL27 cells by tail vein injection. The red arrows show nodules on the lung surfaces. **(B)** Weights of the lungs of BALB/c-nude mice inoculated with OV-NC/OV-CHRDL1 (left) or sh-NC/sh1-CHRDL1 (right) CAL27 cells by tail vein injection. **(C)** Photomicrographs of hematoxylin-and-eosin-stained lungs of mice injected with OV-NC/OV-CHRDL1 (up) or sh-NC/sh1-CHRDL1 (down) CAL27 cells. Error bars represent the standard deviation. *p < 0.05, ***p < 0.001, ****p < 0.0001.

To determine the metastatic effect of CHRDL1 *in vivo*, OV-NC/OV-CHRDL1 and sh-NC/sh1-CHRDL1 CAL27 cells were injected into 4-week-old male BALB/c nude mice *via* the tail vein, and their lung colonization ability was analyzed. Samples were collected after 9 weeks to determine the number of pulmonary tumor colonies and weight changes. As shown in **Figure 6A**, the number of lung tumors (red arrow) derived from OV-CHRDL1 CAL27 cells was significantly decreased compared to that derived from OV-NC CAL27 cells. The number of lung tumors (red arrow) derived from sh1-CHRDL1 CAL27 cells was significantly increased compared to that derived from sh-NC CAL27 cells. The lung weights of the mice injected with OV-CHRDL1 CAL27 cells were lower than those of the mice injected with OV-NC CAL27 cells. In contrast, the lungs from the mice injected with sh1-CHRDL1 CAL27 cells weighed more than those of the mice injected with sh-NC CAL27 cells (**Figure 6B**). Furthermore, hematoxylin-eosin staining of lung tissues showed that mice injected with OV-CHRDL1 CAL27 cells had fewer lung colonies, while mice injected with sh1-CHRDL1 CAL27 cells showed more lung colonies, compared to the control group (**Figure 6C**). These results demonstrated that CHRDL1 silencing could promote tumor metastasis and enhance lung colonization *in vivo*, while CHRDL1 overexpression could restrain tumor metastasis and enhance lung colonization *in vivo*.

## Discussion

The roles of CHRDL1, a BMPS antagonist associated with various cancers, should be more deeply explored. In the present study, we focused on the effect of CHRDL1 on OSCC *in vitro* and *in vivo* and further revealed its function and potential mechanisms.

The expression of CHRDL1 is dysregulated in different types of tumors. CHRDL1 was reported to be downregulated in T-ALL (35), gastric cancer (29), thyroid cancer (31–34), lung cancer (25–28), malignant melanoma (36), and breast cancer (22–24). However, Mock etal. (42) reported an upregulation of CHRDL1, which is activated by the transcription factor ZEB1 and correlated with bone metastasis rather than brain or lung metastasis, in breast cancer. Cha etal. (37) used array-based comparative genomic hybridization (aCGH) and multiplex ligation-dependent probe amplification (MLPA) to screen human genome-wide alterations in seven OSCC tissues and their resection margins. They found that CHRDL1 was one of the eleven genes with the highest amplification frequencies. No additional expression analysis was performed. However, we found lower CHRDL1 expression in OSCC tissues than in adjacent tissues by bioinformatic analysis, RT-qPCR and WB confirmation. The inconsistent findings regarding the expression of CHRDL1 in OSCC might be attributable to various factors, including differences in controls and populations, small sample sizes, and the heterogeneity of OSCC. The GEPIA database (43) was searched to analyze CHRDL1 expression between the tumor and normal groups (**Supplementary Figure 2**). CHRDL1 was downregulated in head and neck squamous cell carcinoma (HNSC), which was consistent with our results. Furthermore, CHRDL1 was also downregulated in esophageal carcinoma (ESCA). It is possible that HNSCC (including OSCC) and ESCA share a common histological origin (i.e., stratified squamous epithelium) (44) and a similar genomic characterization (45–47). CHRDL1 is downregulated in most malignant tumors (except for thymoma), despite their differing histologic origins. We also found that CHRDL1 expression was decreased in OSCC. These findings suggest that CHRDL1 downregulation may regulate the initiation of malignant tumors across different tissues.

CHRDL1 knockdown significantly facilitated migration, invasion, and adhesion, but not proliferation, in OSCC cells. CHRDL1 overexpression reversed these cellular phenotypes. It has been demonstrated that both endogenous CHRDL1 and recombinant CHRDL1 suppress the migration and invasion induced by BMP4 signaling in different breast cancer cell lines (21). CHRDL1 knockdown promotes the proliferation and migration of gastric cancer cells through BMPR II by activating Akt, Erk, and β-catenin (29). Mithani etal. (36) demonstrated growth-suppressive properties with CHRDL1 transfection into melanoma-derived cell lines. Tumor metastasis *in vivo* can be simplified into an ordered process consisting of local invasion, intravasation, survival in the circulation, extravasation, and colonization (48). In an *in vivo* nude mouse model, CHRDL1 knockdown in CAL-27 cells significantly increased the weight of the lungs and the metastatic nodules of the lungs. Overexpression of CHRDL1 reversed this effect.

The initiation of cancer cell metastasis is highly associated with the aberrant activation of the EMT process, which allows cancer cells to disseminate from the primary tumor to the surrounding tissues (49). EMT involves the decreased expression of epithelial markers such as E-cadherin and cytokeratin 18 and the increased expression of mesenchymal markers such as fibronectin and N-cadherin (50). Activation of EMT was also found in gastric cancer and breast cancer cells after CHRDL1 silencing (29, 42). It is worth noting that we observed an EMT-like phenotype in CHRDL1-depleted CAL27 cells.

The dysregulated signaling pathway after CHRDL1 dysregulation was further explored in CAL-27 cells. Mitogen-activated protein kinase (MAPK) signaling pathway members, including p38, ERK1/2, and JNKs, regulate all critical phases of cell growth, including proliferation, differentiation, and apoptosis. The deregulation of the MAPK pathway has been reported in several types of tumors, including human epithelial carcinogenesis, prostate cancers, renal cell carcinoma, hepatocellular carcinoma, glial neoplasms, and breast cancer (51).

Various lines of evidence in OSCC have reported that the phosphorylated activation of p38 promotes cell proliferation, migration, and invasion (52–55). In addition, inhibition of p38 signaling reduces proliferation, angiogenesis, lymphangiogenesis and tumor-induced inflammation (52, 56) and increases apoptosis and autophagy in OSCC tumor cells (57). ERK1/2 phosphorylation appears to promote OSCC cell progression, migration, proliferation, and metastasis (58, 59). Notably, the role of JNK signaling in OSCC is complex and remains controversial. Some authors have argued that downregulation of JNK1/2 pathways inhibits OSCC cell metastasis (60–63) and increases apoptosis and autophagy (57). In contrast, some reports have concluded that reducing the phosphorylation of JNK promotes OSCC cell progression and migration (64–66), which is consistent with our results.

The effects of CHRDL1 on the MAPK signaling pathway have rarely been reported. We found that silencing CHRDL1 in an OSCC cell line could activate the MAPK signaling pathway, leading to the promotion of malignant behaviors in OSCC. In contrast, overexpression of CHRDL1 in CAL27 cells inhibited the expression of phosphorylated ERK1/2 and p38 and increased the level of phosphorylated JNK, leading to the malignant characteristics of OSCC. Several studies of OSCC have indicated that activation of the MAPK signaling pathway induces EMT to increase cellular malignancy (67–70). In addition, MAPK inhibitors can restore the original morphology of epithelial cells or block the expression of EMT-related factors (71, 72). These findings supported the hypothesis that CHRDL1 is associated with EMT and aggressive behaviors through the MAPK pathway in OSCC. MAPK inhibitors should be used to confirm the involvement of ERK1/2, p38, and JNK in CHRDL1-related phenotypes in future studies.

Furthermore, noninvasive detection of salivary, serum, and plasma factors is an important method for OSCC prediction (73–76). Our study is limited by the fact that CHRDL1 levels were not measured in the saliva and serum of OSCC patients due to the lack of such samples.

## Conclusion

In conclusion, our study revealed the modulatory role of CHRDL1 in the malignant characteristics of OSCC as well as the molecular mechanism. The downregulation of CHRDL1 in OSCC may induce EMT and promote metastasis *via* the MAPK signaling pathway, while upregulation of CHRDL1 could reverse this effect. Our study provides the new perspective that CHRDL1 has possible potential as a molecular target in OSCC therapy.

## Data Availability Statement

The data presented in the study are deposited in the NCBI SRA database, accession number: PRJNA801461.

## Ethics Statement

The studies involving human participants were reviewed and approved by the Ethics Committee of the Stomatology Hospital of Guangzhou Medical University (Approval No. KY2019026). The patients/participants provided their written informed consent to participate in this study. The animal study was reviewed and approved by the Institutional Animal Care and Use Committee of the First Affiliated Hospital of Guangzhou Medical University of China (Approval No. 2017-086).

## Author Contributions

QW, ZZ, and LW contributed to the conception and design of the study. QW, ZZ, MX, XX, AW, TZ, SH, and SX performed the experiments. QW, JWZ, ZP, YQ, JYZ, and HC performed the statistical analysis. QW wrote the first draft of the manuscript. QW, LW, ZZ, and KO critically revised the manuscript. All authors contributed to the article and approved the submitted version.

## Funding

This research was funded by the National Natural Science Foundation of China (Grant No. 31801152) and the University Student Laboratory Open Project of Guangzhou Medical University (Grant No. 2020-27).

## Conflict of Interest

The authors declare that the research was conducted in the absence of any commercial or financial relationships that could be construed as a potential conflict of interest.

## Publisher’s Note

All claims expressed in this article are solely those of the authors and do not necessarily represent those of their affiliated organizations, or those of the publisher, the editors and the reviewers. Any product that may be evaluated in this article, or claim that may be made by its manufacturer, is not guaranteed or endorsed by the publisher.
