# Supplementary material for: Chordin-Like 1 Regulates Epithelial-to-Mesenchymal Transition and Metastasis via the MAPK Signaling Pathway in Oral Squamous Cell Carcinoma
Source: Front Oncol. 2022 Apr 14;12:862751. doi: 10.3389/fonc.2022.862751 (PMC9046701; doi:10.3389/fonc.2022.862751)
Supplement: Supplementary file 1 [file DataSheet_1.docx]

**Supplementary Material**

**S1** **Materials and methods**

Table S1
RT qPCR primers used in this study

| Genes | Forward Primers (5–3′) | Reverse Primers (5–3′) |
| --- | --- | --- |
| *GAPDH* | GGAGCGAGATCCCTCCAAAAT | GGCTGTTGTCATACTTCTCATGG |
| *CHRDL1* | GATGGAGAACTGTCATGGGAAC | GGAGGATCATAGTGAGAGCGG |

**Supplementary Figure 1**

**
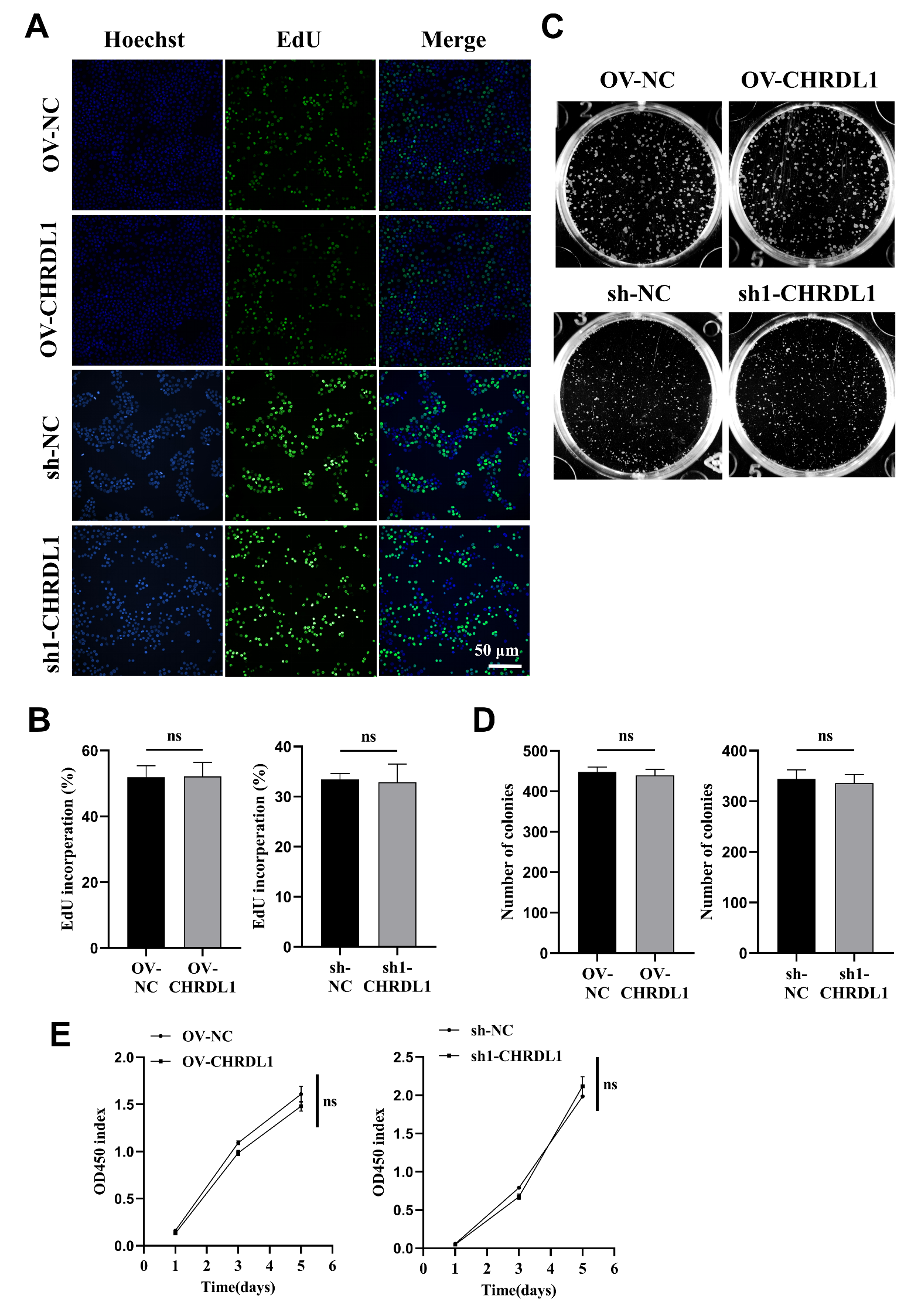
**

**Supplementary Figure 1. CHRDL1 has no Effects on CAL27 cells proliferation. (A-B)** Representative fluorescent images of EdU assays for transfected CAL27 cells and statistical results. **(C-D)** Colony formation assays for transfected CAL27 cells. **(C)** The representative staining images of each group; **(D)** The number of clones formed in each group. **(E)** CCK8 assays for transfected CAL27 cells. Error bars represent the standard deviation. ns = no significance.

**Supplementary Figure 2**


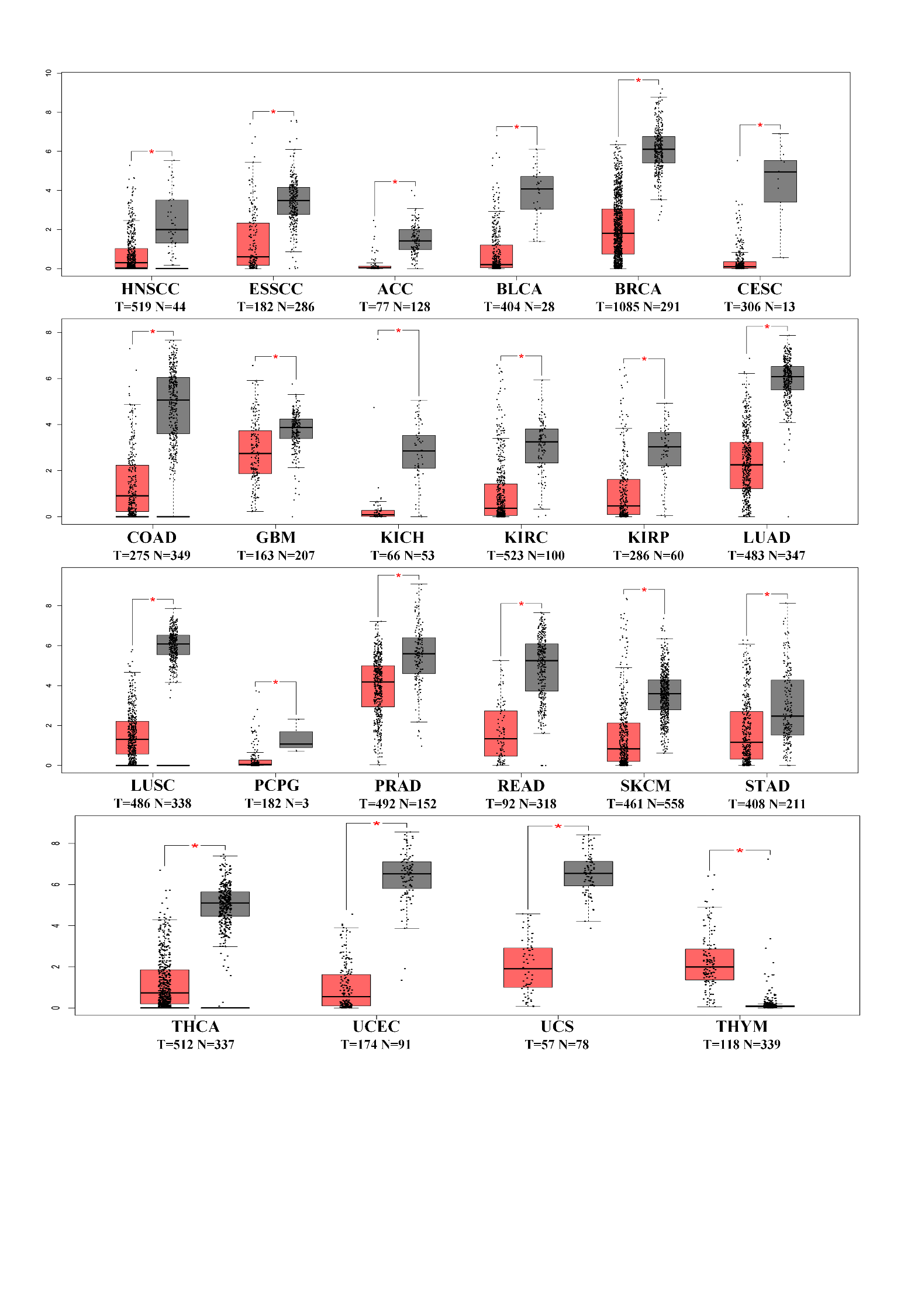


**Supplementary Figure 2. The expression of CHRDL1 in common tumor types.** Tumor types with differential expression of CHRDL1 between tumor groups and normal groups (|Log_2_FC|>1; p <0.05); Data from GEPIA (Gene Expression Profiling Interactive Analysis, http://gepia.cancer-pku.cn/ ); HNSC, Head And Neck Squamous Cell Carcinoma; ESCA, Esophageal Carcinoma; ACC, Adrenocortical carcinoma; BLCA, Bladder Urothelial Carcinoma; BRCA, Breast invasive carcinoma; CESC, Cervical Squamous Cell Carcinoma; COAD, Colon Adenocarcinoma; GBM, Glioblastoma Multiforme; KICH, Kidney Chromophobe; KIRC, Kidney renal clear cell carcinoma; KIRP, Kidney renal papillary cell carcinoma; LUAD, Lung adenocarcinoma; LUSC, Lung squamous cell carcinoma; PCPG, Pheochromocytoma and Paraganglioma; PRAD, Prostate adenocarcinoma; READ, Rectum Adenocarcinoma; SKCM, Skin Cutaneous Melanoma; STAD, Stomach Adenocarcinoma; THCA, Thyroid carcinoma; UCEC, Uterine Corpus Endometrial Carcinoma; UCS, Uterine Carcinosarcoma; THYM, Thymoma.
